# Percutaneous ultrasound gastrostomy (PUG): first prospective clinical trial

**DOI:** 10.1007/s00261-021-03200-x

**Published:** 2021-07-09

**Authors:** Fabio Accorsi, Jonathan Chung, Amol Mujoomdar, Daniele Wiseman, Stewart Kribs, Derek W. Cool

**Affiliations:** Department of Medical Imaging, Schulich School of Medicine and Dentistry, Western University, London, ON Canada

**Keywords:** Ultrasonography, Gastrostomy, Percutaneous ultrasound gastrostomy

## Abstract

**Purpose:**

To report the results of the first-in-human trial evaluating the safety and efficacy of the percutaneous ultrasound gastrostomy (PUG) technique.

**Methods:**

A prospective, industry-sponsored single-arm clinical trial of PUG insertion was performed in 25 adult patients under investigational device exemption (mean age 64 ± 15 years, 92% men, 80% inpatients, mean BMI 24.5 ± 2.7 kg/m^2^). A propensity score-matched retrospective cohort of 25 patients who received percutaneous radiologic gastrostomy (PRG) was generated as an institutional control (mean age 66 ± 14 years, 92% men, 80% inpatients, mean BMI 24.0 ± 2.7 kg/m^2^). Primary outcomes included successful insertion and 30-day procedure-related adverse events (AE’s). Secondary outcomes included procedural duration, sedation requirements, and hospital length of stay.

**Results:**

All PUG procedures were successful, including 3/25 [12%] performed bedside within the ICU. There was no significant difference between PUG and PRG in rates of mild AE’s (3/25 [12%] for PUG and 7/25 [28%] for PRG, *p* = 0.16) or moderate AE’s (1/25 [4%] for PUG and 0/25 for PRG, *p* = 0.31). There were no severe AE’s or 30-day procedure-related mortality in either group. Procedural room time was longer for PUG (56.5 ± 14.1 min) than PRG (39.3 ± 15.0 min, *p* < 0.001). PUG procedure time was significantly shorter after a procedural enhancement, the incorporation of a Gauss meter to facilitate successful magnetic gastropexy. Length of stay for outpatients did not significantly differ (2.4 ± 0.5 days for PUG and 2.6 ± 1.0 days for PRG, *p* = 0.70).

**Conclusion:**

PUG appears effective with a safety profile similar to PRG. Bedside point-of-care gastrostomy tube insertion using the PUG technique shows promise.

*Trial Registration Number*: ClinicalTrials.gov ID NCT03575754.

**Graphical abstract:**

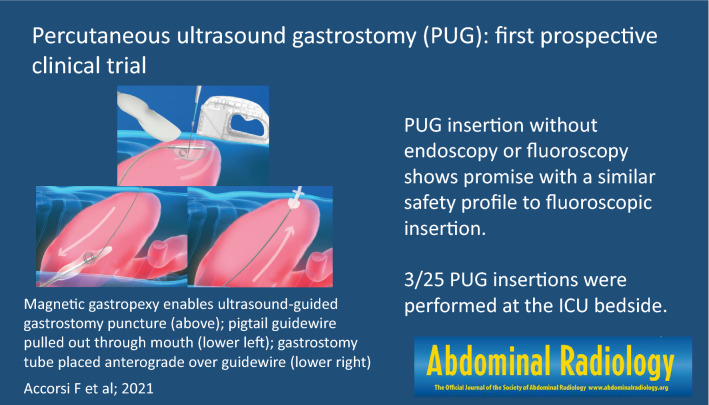

**Supplementary Information:**

The online version contains supplementary material available at 10.1007/s00261-021-03200-x.

## Introduction

Percutaneous gastrostomy tube insertion involves the placement of a feeding tube through the abdominal wall directly into the stomach for enteral nutrition in individuals unable to tolerate per-oral intake. Gastrostomy insertion is a common procedure with nearly 140,000 new insertions performed each year in just the United States Medicare population [1]. Common indications for gastrostomy tube insertion include ischemic or traumatic neurologic injury, head and neck cancer, esophageal cancer, recurrent aspiration, and other reasons for a failed swallowing assessment. This common procedure is most often performed in one of two ways: with the assistance of upper endoscopy (percutaneous endoscopic gastrostomy; PEG) or under fluoroscopic guidance (percutaneous radiologic gastrostomy, or PRG, also sometimes referred to as radiologically inserted gastrostomy, or RIG). Both techniques require specialized equipment and are typically performed in dedicated procedural suites, competing for room time with other endoscopic and fluoroscopic procedures. CT guidance has been suggested for some complex gastrostomy tube insertions, which competes with diagnostic scans [2].

Percutaneous ultrasound gastrostomy (PUG) is a new technique that allows for gastrostomy tube insertion using only ultrasound guidance [3]. The PUG technique incorporates a newly developed technology that uses an external handheld magnet placed over the epigastrium to draw a magnet-tipped orogastric balloon to the anterior stomach. This facilitates ultrasound-guided gastrostomy tract creation for placement of a standard push-type per-oral gastrostomy tube. The PUG technique may allow for gastrostomy tube insertion outside of an endoscopic or angiographic suite, in either a routine procedure room or at point-of-care bedside, such as in the ICU.

This first-in-human pilot trial of percutaneous ultrasound gastrostomy aims to establish the initial safety and efficacy of the PUG technique, using a matched retrospective group receiving standard PRG as an institutional control.

## Methods

Research ethics board approval and investigational device exemption were obtained to perform a prospective, single-arm, non-blinded feasibility, and safety clinical trial of the PUG technique with comparison to a propensity score-matched retrospective control cohort receiving standard PRG (*ClinicalTrials.gov* ID NCT03575754). This industry-sponsored first-in-human clinical research trial was conceived by the sponsor (CoapTech, Baltimore, MD); however, all data collection and analyses were performed locally by the authors without sponsor oversight. The writing of all drafts of the manuscript and decision to publish were done by the authors alone. None of the authors have a financial relationship with the sponsor or any other relevant conflicts of interest.

All adults ≥ 18 years of age referred to the interventional radiology department with an indication for gastrostomy tube insertion between October of 2018 and April of 2020 were screened for enrollment in the prospective PUG group. Exclusion criteria included a history of prior gastrostomy tube; BMI < 20 kg/m^2^ or > 30 kg/m^2^; untreated esophageal cancer or esophageal stricture; untreated or recently resected head and neck cancer; prior major upper abdominal surgery or suboptimal positioning of the stomach based on any available prior imaging; other esophageal, head and neck, or upper abdominal condition or anatomy considered by the operator to preclude safe insertion of the orogastric balloon; active life-threatening hemorrhage, hematocrit < 0.25, or blood transfusion within the preceding 14 days; temperature > 38 °C, systolic blood pressure < 100 mmHg or > 180 mmHg, or heart rate < 50 BPM or > 100 BPM; contraindication to being placed in proximity to a magnet, such as possessing a pacemaker; pregnant or nursing women; and individuals in which sedation is contraindicated, such as those with bulbar palsy or amyotrophic lateral sclerosis. The decision to pursue gastric feeds as opposed to post-pyloric feeds was made by the referring primary care teams.

**Fig. 1:**
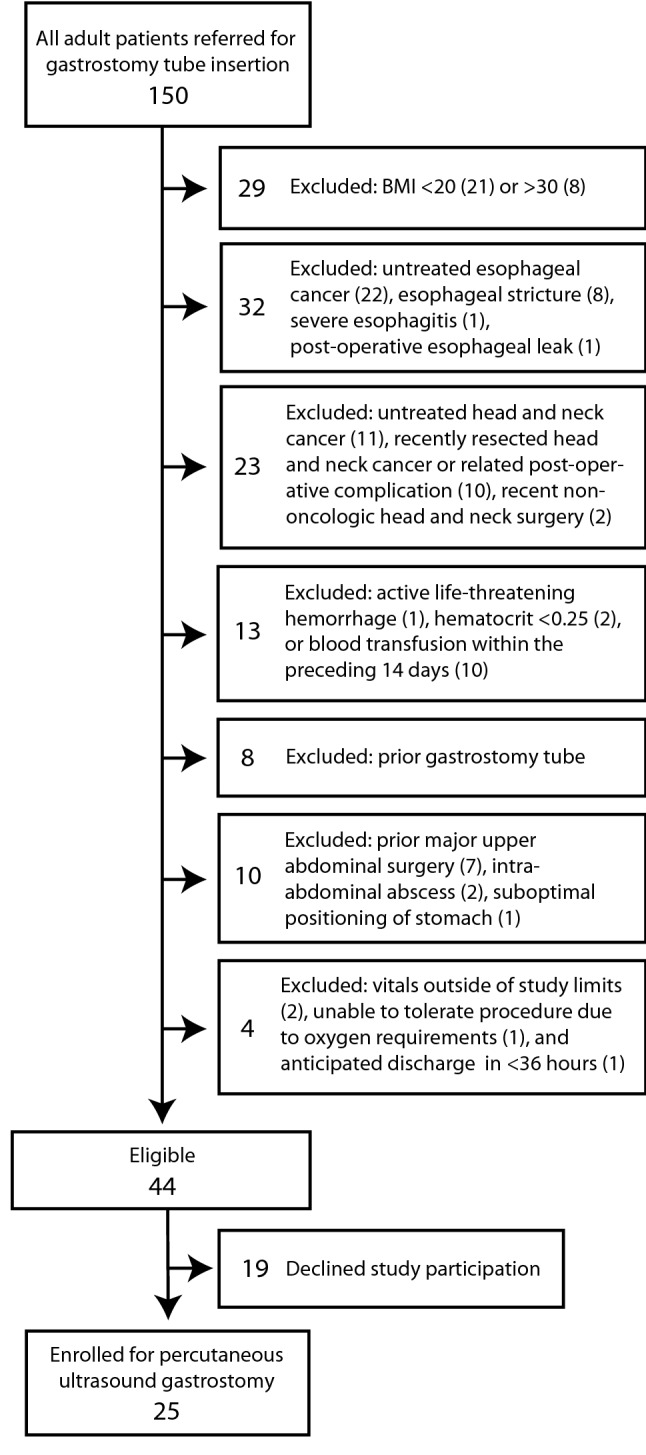
Breakdown of excluded and enrolled individuals. The number of individuals meeting each specific criterion within a category is shown in parentheses. Thirteen individuals met two exclusion criteria simultaneously

Of 150 consecutive patients screened, on whom exclusion criteria were applied, 25 participants were enrolled for percutaneous ultrasound gastrostomy (Fig. 1). A sample size of 25 was selected to establish the first-in-human early safety and efficacy data for the new technique. The first five participants of this study were previously included in a brief report describing the PUG procedure technique [3]. The current study reports on the full clinical trial with quantitative analysis of the safety and efficacy measures and includes comparison to a matched historical cohort receiving PRG.

### Procedural technique

The setting for the PUG insertion (bedside vs. IR department) was determined by the patient’s hospital location. PUG procedures were performed bedside within the ICU for all patients located in the ICU, while PUG procedures for all other patients were performed in an angiographic suite. Portable fluoroscopy was not made available for cases performed bedside within the ICU. The PUG procedure has been previously described [3], is summarized below, and is depicted in Video 1. PUG was performed using a Point-of-Care Ultrasound Magnetically Aligned Gastrostomy kit (PUMA-G System, CoapTech LCC, Baltimore, MD), which includes a reusable handheld external magnet, a single-use orogastric balloon catheter with a bar magnet at the tip, and a coil-tipped guidewire. PUG insertions were performed by one of two interventional radiologists with procedure assistance from the scrub nurse and the circulating technologist that are routinely part of IR procedures.

**Fig. 2:**
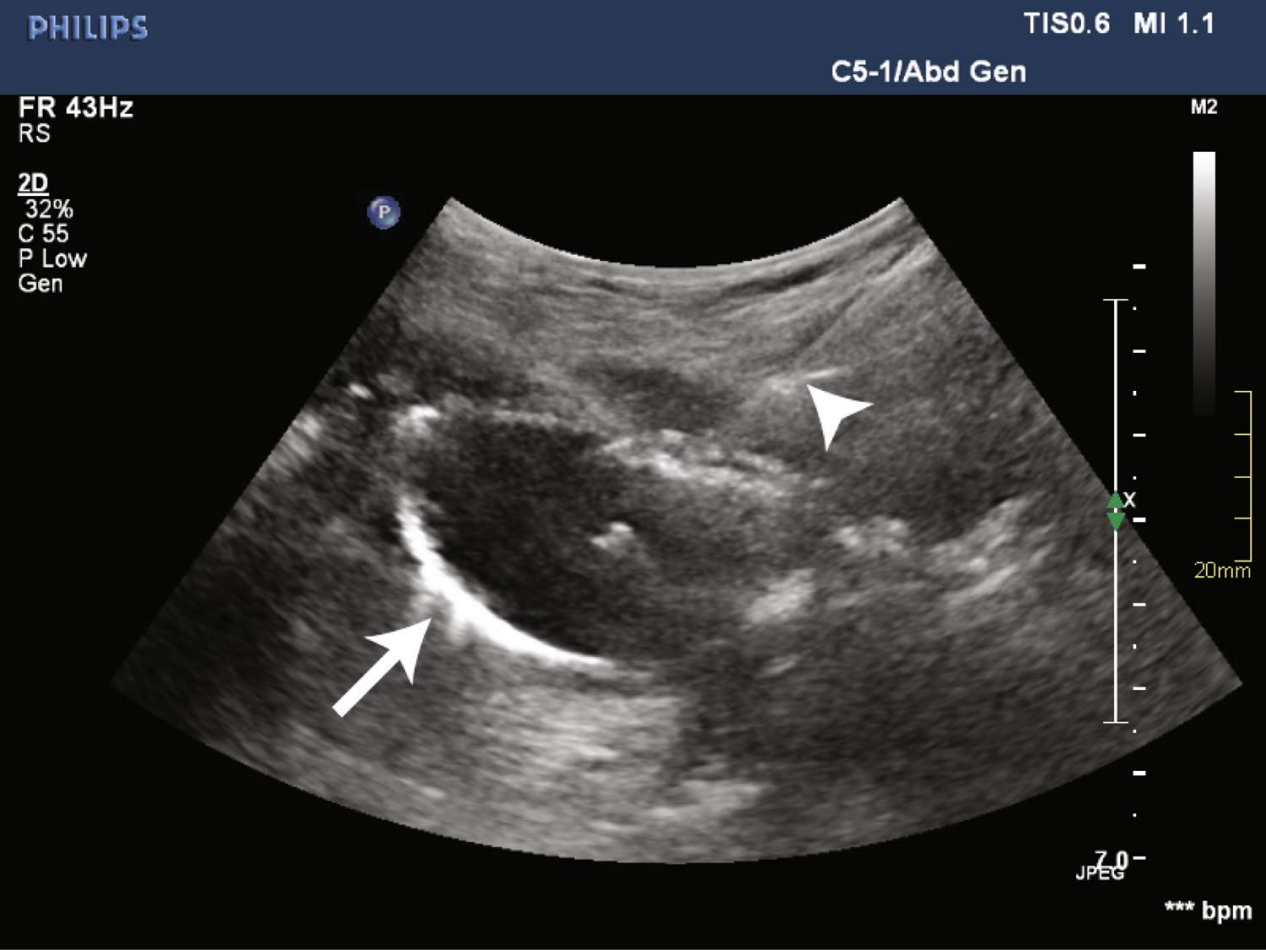
Transabdominal ultrasound of the inflated orogastric balloon (arrow) within the gastric lumen. The orogastric balloon is being pulled against the anterior gastric wall via magnetic gastropexy. An 18-gauge needle (arrowhead) is advanced through the abdominal wall into the orogastric balloon, creating the gastrostomy tract

Titrated doses of Fentanyl and Midazolam were used in all procedures to achieve moderate sedation. The oral cavity was anesthetized with topical lidocaine spray and, if not already in place, a nasogastric tube was inserted to insufflate the stomach. The orogastric balloon was inserted through the mouth into the stomach in a non-sterile manner by the interventional radiologist, and the handheld magnet was placed over the epigastrium by the assistant, drawing the balloon catheter against the anterior gastric wall and achieving magnetic gastropexy. For the purposes of this study, if magnetic coaptation was not achieved, fluoroscopy could be used at the operator’s discretion to locate the orogastric balloon within the stomach and aid in magnetic coaptation. If magnetic gastropexy was not achieved at the bedside within the ICU, the procedure would be rescheduled within a fluoroscopic suite. Once magnetic coaptation was achieved, the interventional radiologist scrubbed in to perform the epigastric ultrasound-guided gastrostomy puncture in a sterile fashion. The orogastric balloon was inflated with 20–30 ml of saline by the assistant, allowing it to be visualized with ultrasound through the abdominal wall. Using real-time ultrasound guidance, an 18-gauge needle was used to puncture the balloon through the abdominal wall to create the gastrostomy tract (Fig. 2). The provided guidewire was inserted through the needle and uncoiled in, or through, the balloon. The balloon was deflated by the technologist to mechanically capture the guidewire, allowing the wire to be drawn out of the mouth by the interventional radiologist, leaving a ‘through-and-through’ guidewire from mouth to gastrostomy site. A 20-French bumper style gastrostomy tube (FLOW 20® PUSH PEG, Cook Medical, Bloomington, IN) was then fed through the guidewire using Sacks-Vine per-oral gastrostomy insertion technique [4].

Two protocol deviations for the PUG procedure occurred during the study. Firstly, pre-procedural prophylactic Cefazolin 1 g IV was administered commencing with the seventh enrolled individual. Administration of prophylactic antibiotics was added to adhere to recommendations for PEG insertion [5], on the basis that both PEG and PUG require advancement of the gastrostomy tube through the mouth and esophagus, increasing the risk of infection with oral microflora at the gastrostomy site. Conversely, institutional practice does not routinely administer antibiotic prophylaxis for PRG and no consensus exists for its use in SIR guidelines [6]. Second, a reusable Gauss meter (Gauss Meter Model VGM, AlphaLab Inc., Salt Lake City, UT) was incorporated into the PUG procedure starting with the tenth participant to assist with the challenge of locating the orogastric balloon within the stomach without fluoroscopic assistance. This handheld meter measures magnetic force with a small directional detector, which was manipulated over the epigastrium during the insertion of the orogastric balloon catheter to detect the position of the balloon’s magnet within the stomach and assist in magnetic coaptation/gastropexy.

PRG insertions were performed with either 12-French Cope-loop multipurpose catheters in 21/25 cases (84%) (Cook Medical, Bloomington, IN; or Boston Scientific, Marlborough, MA) or with 14-French balloon-retention gastrostomy feeding tubes in 4/25 cases (16%) (Avanos, Alpharetta, GA). The type of PRG tube used was primarily based on operator preference. De novo insertion of PRG tubes larger than 14F was not part of the institutional or regional practice. Conversely, the smallest caliber per-oral PEG tube available within the institute was 20F. Hence, a discrepancy in gastrostomy tube size could not be controlled with the propensity match. Specific indications for gastrostomy insertion in each group are outlined in Table 1. PRG insertions were performed by one of six operators, including the two operators involved in PUG insertion.

**Table 1:** Indications for gastrostomy tube insertion within the PUG and PRG groups

Category	PUG (number enrolled)	PRG (number of controls)
Head and neck cancer	Head and neck cancer (9)	Head and neck cancer (10)
Ischemic neurologic impairment	Stroke (4)	Stroke (5) Global anoxic brain injury (2)
Non-ischemic neurologic impairment	Traumatic brain injury (3) Traumatic cervical spine injury (3) Epidural abscess (1) Malignant catatonia (1)	Traumatic cervical spine injury (2) Autoimmune encephalitis (1) Hydrocephalus and meningitis (1) Cervical spondylotic myelopathy (1)
Recurrent aspiration	Recurrent aspiration (2)	Recurrent aspiration (1)
Failed swallowing assessment (not otherwise specified)	Failed swallowing assessment (2)	Failed swallowing assessment (2)

### Follow-up

Participants were maintained NPO with no enteral feeding for 24-h post-insertion. Following the established institutional practice for PRG, all outpatients were admitted post-PUG insertion under the care of the referring service and were discharged by the referring service after nutritional targets were met (typically resulting in a 48-h admission). Clinical follow-up was performed by an interventional radiologist at 48-h and 30-day post-procedure.

### Outcomes

Primary outcomes included procedural success (defined by insertion of a gastrostomy tube into the stomach lumen and achievement of target nutrition) and 30-day procedure-related adverse events (defined by the 2017 SIR adverse event classification system for interventional radiology procedures) [7] (Table 2).

**Table 2:** Society of interventional radiology adverse event classification for interventional radiology procedures [7]

**Mild AE**: No therapy or nominal (nonsubstantial) therapy (postprocedural imaging performed and fails to show manifestation of AE); near miss (e.g., wrong site of patient prepared, recognized and corrected before procedure, wrong patient information entered for procedure)
**Moderate AE**: Moderate escalation of care, requiring substantial treatment, e.g., intervention under conscious sedation, blood product administration, extremely prolonged outpatient observation, or overnight admission after outpatient procedure not typical for the procedure (excludes admission or hospital days unrelated to AE)
**Severe AE:** Marked escalation of care, i.e., hospital admission or prolongation of existing hospital admission for > 24 h, hospital admission that is atypical for the procedure, inpatient transfer from regular floor/telemetry to intensive care unit, or complex intervention performed requiring general anesthesia in previously nonintubated patient (generally excludes pediatrics or in circumstances in which general anesthesia would primarily be used in lieu of conscious sedation, e.g., in mentally challenged or severely uncooperative patients)
**Life-threatening or disabling event**, e.g., cardiopulmonary arrest, shock, organ failure, unanticipated dialysis, paralysis, and loss of limb or organ
**Patient death or unexpected pregnancy abortion**

Secondary outcomes include sedation requirements (utilization and dose of Fentanyl and Midazolam), hospital length of stay (only for outpatients admitted specifically for gastrostomy tube insertion), and procedural duration.

Duration of PUG insertion was prospectively recorded by an independent technologist within the room. *Length of procedure* was defined as the time from pre-procedural ultrasound (start of procedure) until the gastrostomy tube was successfully in place. *Time to magnetic gastropexy* was defined as the time from the start of procedure to magnetic coaptation. *Procedural room time* for PUG was defined as the time from patient entry to patient egress from the procedural room. *Procedural room time* for PRG was recorded in the electronic medical record, as for all interventional procedures, commencing with patient entry into the procedure room and terminating when the study was completed by the technologist in the electronic medical record.

### Control matching

Potential controls were identified by retrospective chart review of all adult PRG procedures performed at the same center in the nine months prior to the first PUG insertion. The same inclusion and exclusion criteria used for the PUG group were applied to the retrospectively identified cohort receiving PRG. A database of 55 potential institutional controls was generated.

Propensity score matching was performed using ‘R: A language and environment for statistical computing’ [8] employing the statistical package ‘MatchIt: Nonparametric Preprocessing for Parametric Causal Inference’ [9]. Propensity scores were generated for age, sex, and inpatient status, felt to be the covariates most likely to affect selection for PUG versus PRG in a routine clinical setting. The ‘optimal matching’ method was used. Propensity score matching resulted in a well-matched group of twenty-five individuals who received PRG, with similar demographic distribution to the PUG group (Table 3).

**Table 3:** Demographic information for the PUG treatment group and PRG control group

Demographic	PUG treatment group	PRG control group	*p* value
Mean age (years)	64 ± 15	66 ± 14	0.61
Men	23/25 (92%)	23/25 (92%)	1
Inpatient Status	20/25 (80%)	20/25 (80%)	1
BMI (kg/m^2^)	24.5 ± 2.7	24.0 ± 2.7	0.52

There was no significant difference in mean age, sex, inpatient status, or BMI

### Statistical analysis

Statistical differences in outcomes between the treated and control groups were evaluated with standard t tests for outcomes with continuous variables (e.g., sedative dose) and two proportion z tests for outcomes with categorical variables (e.g., adverse events). To account for the problem of multiple comparisons and control for familywise error, the significance level (alpha) was adjusted where applicable using the Holm–Bonferroni correction method and explicitly stated in the text as a ‘corrected alpha.’ A starting alpha of 0.05 was used. Single comparisons performed on subgroups (e.g., length of stay for outpatients only) use a standard alpha of 0.05. Trends in procedural length throughout the study were tested using the Pearson Product-Moment Correlation method. Margin of error estimations for adverse events referenced in the discussion were performed using standard formulas for sample size determination for two independent variables with dichotomous outcomes.

## Results

**Fig. 3:**
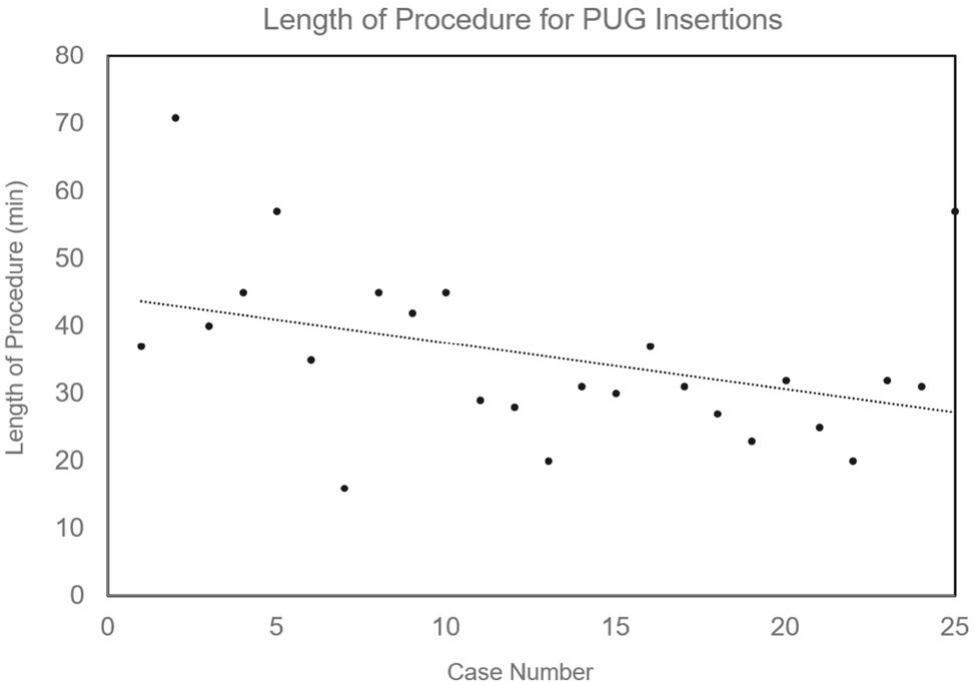
Trend in length of procedure for PUG insertions. Procedure numbers displayed on the x-axis are in chronological order. Pearson Product-Moment Correlation shows a weak negative relationship (r = − 0.395)

Technical success rate was 100% (25/25) for both PUG and PRG. Three (3/25) PUG procedures were performed in the ICU (12%) and 22/25 in the angiographic suite (88%). No PUG procedures performed within the ICU had to be rescheduled in a fluoroscopic suite because of difficulties achieving magnetic gastropexy. There was no difference in the mean length of procedure between the angiographic suite (36.3 ± 13.1 min) and ICU (29.0 ± 1.6 min, *p* = 0.35). There was a weak trend toward reduced length of procedure as operators became more familiar with the technique (r = − 0.395, Fig. 3). Fluoroscopy was used in 8/25 (32%) PUG insertions to assist in locating and coapting the orogastric balloon. Mean length of procedure was longer when fluoroscopy was required (45.6 ± 12.7 min) than when it was not (30.7 ± 9.2 min, p = 0.003). Beginning with the tenth case, a Gauss meter was introduced to assist magnetic coaptation, which was associated with a statistically significant reduction in mean length of procedure from 43.1 ± 14.2 min to 31.1 ± 9.0 min (*p* = 0.016, corrected ɑ = 0.025) and mean time to magnetic gastropexy (32.7 ± 14.0 min to 19.1 ± 4.2 min, p = 0.001, corrected ɑ = 0.017). Rates of fluoroscopy use trended lower when a Gauss meter was employed (3/16 PUG insertions, 19%) than when it was not (5/9 PUG insertions, 56%, *p* = 0.06, ɑ = 0.05). Of the sixteen cases utilizing a Gauss meter, fluoroscopy was used in two of the first three and only one of the last thirteen (8%). Mean procedural room time was longer for PUG insertion (56.5 ± 14.1 min) than for PRG controls (39.3 ± 15.0 min, p < 0.0001, corrected ɑ = 0.01), even after a Gauss meter was introduced (52.3 ± 10.4 min, p = 0.004).

Three (12%) mild procedure-related adverse events occurred after PUG insertion, one mild aspiration and two local stoma site infections, each managed with antibiotics. One (4%) moderate PUG-related adverse event occurred, an abdominal wall abscess near the gastrostomy site managed with percutaneous drainage. The abscess and one of the mild local stoma site infections developed early in the study in patients whom did not receive prophylactic antibiotics. Adverse event rates did not significantly differ from the historical PRG cohort, in which seven mild adverse events (28%, *p* = 0.16, corrected ɑ = 0.025) and no moderate adverse events (*p* = 0.31, corrected ɑ = 0.05) occurred. The mild PRG-related adverse events included two mild aspirations treated with antibiotics and five premature tube failures requiring tube exchange in the angiographic suite without sedation (1 blocked multipurpose tube, 1 blocked balloon-retention tube, and 3 dislodged multipurpose tubes). There were no severe adverse events in either the PUG or PRG group. There were no reports of oropharyngeal pain during the follow-up period after PUG insertion. No significant difference in mean length of procedure was observed between PUG insertions with adverse events (42.3 ± 9.8 min) and those without complication (34.1 ± 12.6 min, *p* = 0.23). Rates of fluoroscopy use were statistically significantly higher for PUG insertions with adverse events (3/4 [75%]) than those without (5/21 [24%], *p* = 0.04).

A single mortality was observed in the PUG group, occurring 12 days post-gastrostomy tube insertion secondary to aspiration pneumonia. This individual was a 93-year-old with significant comorbidities, including recurrent aspirations, in whom gastrostomy procedure was performed without immediate complication. On the eighth day post-procedure, the patient experienced an acute aspiration event, confirmed radiographically as new bibasilar consolidation, with subsequent decline in his clinical status, resulting in death on the twelfth day post-procedure. The aspiration event and mortality were deemed to be unrelated to the PUG insertion procedure by an independent data safety monitoring board comprised of a gastroenterologist specializing in endoscopic procedures. No PUG-related 30-day mortality was recorded. There was no PRG-related 30-day mortality.

Sedation was used in 25/25 (100%) PUG insertions and 17/25 (68%) PRG procedures. The lower rate of sedation use in the PRG group was attributed to the sedation practices of one of the interventionists in the group who was not involved in the PUG insertions. This interventionist performed 10 PRG procedures, 8 of which were done without sedation. Mean dose of Midazolam for all-comers, regardless of whether sedation was intended or not, was higher for the PUG group (2.28 ± 1.37 mg; median dose 2 mg) than for PRG controls (1.28 ± 1.41 mg, *p* = 0.014, corrected ɑ = 0.017; median dose 1 mg), as was the mean dose of Fentanyl for all-comers (110 ± 70.7mcg for the PUG group and 54.0 ± 58.6mcg for PRG controls, *p* = 0.004, ɑ = 0.013; median doses of 100mcg and 50mcg, respectively)**.** However, excluding the eight PRG insertions where sedation was not intended, there was no difference between the groups in the mean dose of Midazolam (2.28 ± 1.37 mg for PUG, 1.88 ± 1.33 mg for PRG, *p* = 0.35) or Fentanyl (110 ± 70.7mcg for PUG, 79.41 ± 55.1mcg for PRG, *p* = 0.14).

Mean hospital length of stay for outpatients admitted to hospital specifically for gastrostomy tube insertion did not differ between the PUG group (2.4 ± 0.5 days, *n* = 5) and PRG controls (2.6 ± 1.0 days, *n* = 5) (*p* = 0.70).

## Discussion

This first-in-human prospective study establishes the feasibility and early safety of the percutaneous ultrasound gastrostomy technique, with successful PUG insertion performed in all 25 participants. No 30-day procedure-related mortality or severe adverse events occurred after PUG insertion. Rates of mild (12%) and moderate (4%) procedure-related adverse events did not significantly differ from those of a matched institutional control receiving PRG. These rates were compared to the reported rates of minor and major adverse events of 5.9–19.5% and 7.4–9.4%, respectively, after PEG [10]. Rates of infection after PUG totaled 12%, slightly higher than those reported in the literature (7% after PRG and 6% after PEG) [11], but within the estimated margin of error of 12% for the sample size of this study. In the outpatient subgroup admitted to hospital specifically for gastrostomy tube insertion, length of hospital stay following PUG insertion did not differ either, suggesting similar procedure-related recovery and time to nutrition.

A potential benefit of the PUG technique is the ability to perform gastrostomy tube insertion outside of the angiographic suite, such as at point-of-care bedside or in a non-fluoroscopy IR procedure room. Moving gastrostomy tube insertion procedures into a general procedure room may permit interventional radiologists to expand their practice capacity by keeping the angiographic suite available for use in other fluoroscopic procedures. Indeed, successful deployment of this technology depends on the ability to reliably perform PUG insertion without fluoroscopic assistance. While only 68% (17/25) of PUG insertions were performed without fluoroscopy, growing operator experience and implementation of a Gauss meter resulted in substantial reduction in fluoroscopy use, such that it was only employed in a single of the last thirteen cases (8%). Importantly, failure to achieve magnetic gastropexy during a bedside PUG insertion would occur before sterile preparation or percutaneous puncture, so the procedure may be easily canceled and rescheduled in a fluoroscopic suite if necessary. Although the low number of PUG insertions performed in the ICU limits conclusions regarding ICU-specific outcomes, all bedside insertions were performed without intra-procedural issues or 30-day procedure-related adverse events.

The need to insert two enteric tubes during PUG insertion (a nasogastric tube for gastric insufflation and the PUMA-G orogastric balloon catheter) is a limitation of PUG which may make it less appealing compared to PRG, particularly in the outpatient setting. This may be remedied in a future iteration of the orogastric balloon catheter by incorporating a second lumen allowing for gastric insufflation and magnetic gastropexy via the single device.

Procedural room occupancy was longer for PUG than for PRG controls, which is likely related to the challenge in PUG procedures of inserting the orogastric balloon catheter and achieving magnetic gastropexy without fluoroscopic guidance, as well as the current relative inexperience with the PUG technique compared to the established PRG. This is supported by the observed trend toward shorter procedural duration with each insertion performed and after a Gauss meter was introduced. It is possible that as operators become increasingly familiar with this new technique, and the steps become more refined, procedural efficiency will continue to improve and the length gap may narrow further.

Excluding procedures where sedation was not intended, there was no significant difference in mean dose of Fentanyl or Midazolam between the groups. This suggests that in practice settings where sedation is routinely employed for gastrostomy tube insertion, PUG and PRG sedation requirements may be equivalent. Notably, as with any per-oral gastrostomy technique, the need for sedation in PUG insertion limits its utility in populations for which sedation is not preferred, such as in those with bulbar palsy (e.g., amyotrophic lateral sclerosis).

The stringent exclusion criteria of this feasibility study resulted in omission of 70% of screened patients (106/150), but real-world eligibility for PUG is expected to be higher. Firstly, some of the exclusionary criteria are considered unlikely to affect the feasibility and safety of PUG, namely having a low hematocrit level or recent blood transfusion but stable clinical status (12 of the screened patients, 8%), having had a prior gastrostomy tube or major upper abdominal surgery (15 patients, 10%), and having a BMI < 20 kg/m^2^ (21 patients, 14%). Simply including these patients would result in an eligibility rate of 53% (79/150) for PUG. Another major contributor to eligibility in this study is the relatively large proportion of patients at this institution referred with upper aerodigestive tract (UADT) malignancies (head and neck or esophageal cancer, 33/150 or 22% of those screened). Patients who had not begun cancer treatment were excluded due to concerns of tumor seeding along the gastrostomy stomal site during advancement of the gastrostomy tube, a complication reported to occur in only 0.5% of patients with UADT malignancies receiving per-oral gastrostomy [12], the impact of which on overall survival is uncertain. The eligible population in a center with a different referral base may be higher.

Eight (8/150) obese patients with a BMI > 30 k/m^2^ (5% of those screened) were excluded based on device specifications related to the limited applied magnetic force of the external magnet on the orogastric balloon when skin-to-stomach distances exceed 5 cm. A stronger external magnet should overcome the magnetic force limitations, but the impact on sonographic balloon visualization and safe gastrostomy tract puncture in larger patients requires separate evaluation. Additionally, the potential risks of placing a strong external magnet near the pacemaker device remain to be clarified to determine if this is a contraindication for PUG insertion.

The relatively small sample size of this pilot study is a limitation, an issue which is compounded in cases where subgroup analyses were necessary (e.g., data relating to cases utilizing a Gauss meter). This limitation is particularly relevant with respect to rare outcomes such as moderate adverse events, found to be 4% for PUG and 0% for PRG in this study. Power calculations reveal that the sample size of 25 yields an estimated margin of error of 7.7% for these outcomes, illustrating that reliable detection of a difference in these rare outcomes requires a larger study. Similar limitations apply to the detection of 30-day procedure-related mortality (reported at 1% for both PRG and PEG [11]). Furthermore, PUG was performed by two interventional radiologists with substantial experience in diagnostic ultrasound interpretation and percutaneous ultrasound-guided needle procedures. The efficacy and safety of the PUG procedure in the hands of non-IR operators should not be assumed. This was a safety and efficacy study for a new device and procedural technique, and as such, was not designed as a randomized control trial. Although efforts were made to control potential confounders in the matching of retrospective controls, some unidentified selection bias may remain. For example, the number of operators involved in the retrospective cohort was larger than those prospectively performing PUG insertion, so operator-dependent factors were not controlled for in the propensity match. Lastly, PUG procedural room times were measured prospectively by an independent technologist as part of the study protocol, whereas PRG procedural room times were retrospectively obtained from EMR records. As such, there may be bias related to inconsistencies or variabilities in the way PRG times are recorded. Nevertheless, this remains the best available data for comparison to this retrospective cohort.

The presented early feasibility and safety data for percutaneous ultrasound gastrostomy support larger-scale, prospective, randomized control trials comparing the PUG technique to established methods such as PRG and PEG. The utility of PUG as a portable method of gastrostomy insertion may prove complementary to these established methods and warrants further investigation to establish the optimal patient populations and role within the interventional radiology practice.

## Supplementary Information

Below is the link to the electronic supplementary material.

Supplementary file1 (MOV 26147 KB)

## Data Availability

Anonymized data may be made available upon request for validation purposes.

## Code Availability

Statistical analysis was performed with ‘R: A language and environment for statistical computing’ employing the statistical package ‘MatchIt: Nonparametric Preprocessing for Parametric Causal Inference.'
